# Supplementary material for: Splice-Junction-Based Mapping of Alternative Isoforms in the Human Proteome
Source: Cell Rep. Author manuscript; Available in PMC 2020 Jan 15. (PMC6961840; doi:10.1016/j.celrep.2019.11.026)
Supplement: 2 [file NIHMS1546469-supplement-2.zip › DF1/PXD010154/Liver/Liver_29_KANK1_TLGEWQKGTMQLILK.pdf]

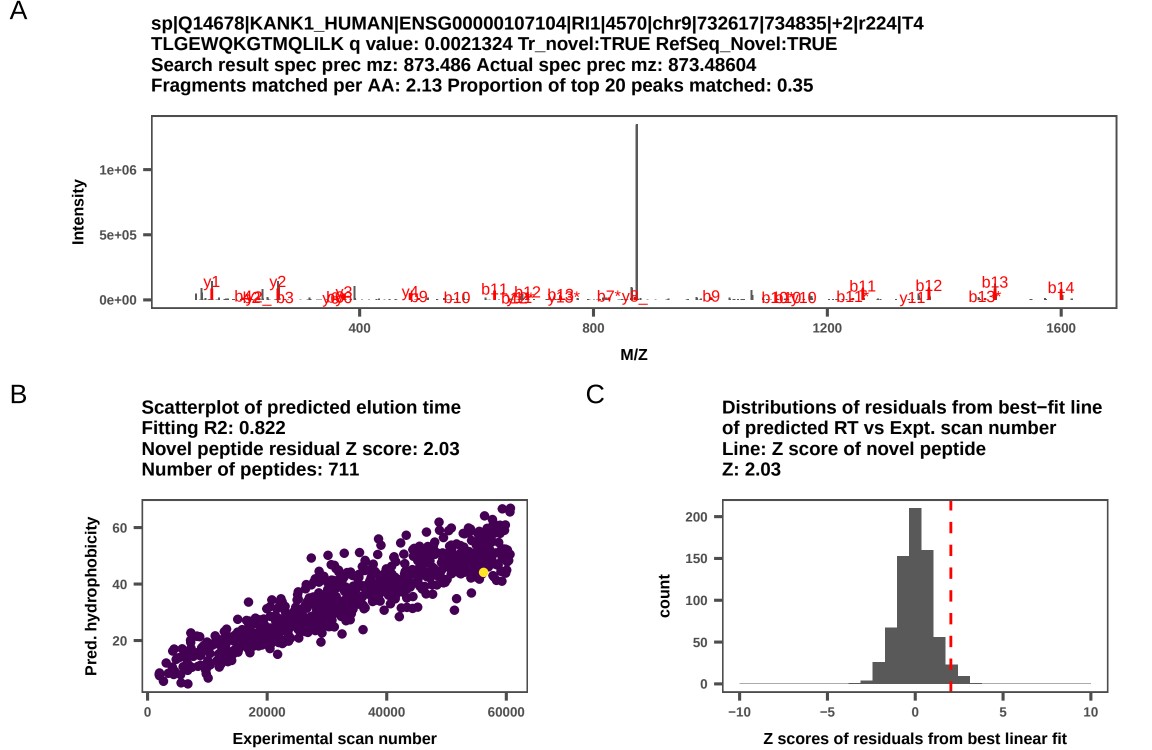
A
sp|Q14678|KANK1_HUMAN|ENSG00000107104|RI1|4570|chr9|732617|734835|+2|r224|T4
TLGEWQKGTMQLILK q value: 0.0021324 Tr_novel:TRUE RefSeq_Novel:TRUE
Search result spec prec mz: 873.486 Actual spec prec mz: 873.48604
Fragments matched per AA: 2.13 Proportion of top 20 peaks matched: 0.35
Intensity
1e+06
5e+05
0e+00
400
800
1200
1600
M/Z
B
Scatterplot of predicted elution time
Fitting R2: 0.822
Novel peptide residual Z score: 2.03
Number of peptides: 711
Pred. hydrophobicity
60
40
20
0
20000
40000
60000
Experimental scan number
C
Distributions of residuals from best−fit line
of predicted RT vs Expt. scan number
Line: Z score of novel peptide
Z: 2.03
count
200
150
100
50
0
−10
−5
0
5
10
Z scores of residuals from best linear fit
